# A new approach to assessing pterygium progression and volume using SII and anterior segment OCT

**DOI:** 10.3389/fmed.2025.1758913

**Published:** 2026-01-14

**Authors:** Rui Niu, Xin Jin, Zhongyang Yan, Ziqi Zhao, Lina Fan, Zhe Du, Lifei Wang, Jingjiang Liu, JunSheng An, Xiaoxuan Wang

**Affiliations:** Hebei Eye Hospital, Hebei Key Laboratory of Ophthalmology, Hebei Provincial Clinical Research Center for Eye Diseases, Xingtai, Hebei, China

**Keywords:** anterior segment OCT, ordered logistic, pterygium, SII, volume

## Abstract

**Purpose:**

To compare the clinical manifestations and prognosis of 90 patients with pterygium. The occurrence and progression of pterygium are associated with the systemic immune-inflammatory index (SII) and clinical characteristics. The size of pterygium was assessed by anterior segment phase and anterior segment optical coherence tomography (AS-OCT).

**Methods:**

According to the pterygium score, the patients were divided into group A, group B, and group C. The evaluation parameters included gender, age, onset time, signs and symptoms of underlying diseases, AS-OCT, Neutrophil-to-Lymphocyte Ratio (NLR), Platelet-to-Lymphocyte Ratio (PLR), Monocyte-to-Lymphocyte Ratio (MLR), and SII values. The postoperative pterygium tissue was weighed. The clinical characteristics of each group were analysed by a nonparametric test and a chi-square test. Ordered logistic regression analysis evaluated the parameters related to pterygium area and volume.

**Results:**

Compared with the control group, the SII, NLR, and MLR values of pterygium patients were significantly increased. The incidence of both eyes in group C was significantly higher than that in groups A and B. The incidence of both eyes and the SII value would affect the severity of pterygium. The SII value was significantly correlated with the area of pterygium progression and weighing volume. The combination of the anterior segment phase and AS-OCT can effectively evaluate the volume of pterygium.

**Conclusion:**

SII can be used as a biomarker to predict the severity and prognosis of pterygium. Compared with traditional methods, imaging data can better evaluate the progression of pterygium by calculating the volume of pterygium.

## Introduction

Pterygium is an ocular surface disease characterized by an invasive fibrovascular lesion from the bulbar conjunctiva to the cornea (1). Its occurrence and progression are related to environmental exposure, genetic susceptibility and chronic inflammation, which are mainly manifested as a series of active pathological processes such as proliferation, inflammatory infiltration, fibrosis, angiogenesis, and extracellular matrix remodeling (2, 3). The clinical harm of this disease is its insidious course and the prediction model of corneal invasion that is not completely accurate. In the early stage, patients often have no subjective symptoms, but when foreign body sensation occurs or its appearance changes, the lesions have mostly invaded the corneal optical zone. Therefore, early evaluation and prediction can help patients to find the right time for surgery, reduce the damage to the cornea, and evaluate the recovery.

The current clinical scoring system of pterygium (the Modified Pterygium Classification System) relies on the observation and experience of doctors, resulting in poor reproducibility of the severity assessment of pterygium (4). Moreover, the scoring focuses on the horizontal extension of the conjunctival tissue to the corneal plane, but fails to effectively quantify the longitudinal invasion depth of the corneal stroma (4, 5).

Through high-resolution tomography, anterior segment optical coherence tomography (AS-OCT) is currently mainly used for the assessment of the Angle of glaucoma, the measurement of corneal morphology and thickness, and the analysis of anterior segment tumors to achieve accurate diagnosis and monitoring (6). The ability of AS-OCT to visualize the anatomical characteristics of pterygium and quantify its invasion of the corneal layer provides objective thickness data, which is of certain significance for assessing the severity of the disease and making treatment decisions (7, 8). Systemic Immune-Inflammation Index (SII) is a composite inflammatory index that integrates platelet, neutrophil, and lymphocyte counts. The SII value of pterygium patients is significantly higher than that of healthy controls, and the combination of systemic inflammation or allergic diseases is regarded as a clear risk factor for pterygium (9–11). The core pathological features of pterygium are neovascularization and chronic inflammation. Platelets release platelet-derived growth factor (PDGF) and other pro-angiogenic factors to induce pathological vascular proliferation (12, 13). At the same time, inflammatory cells such as neutrophils release reactive oxygen species and proteases locally in pterygium tissue, which aggravates tissue damage and abnormal repair (14).

Therefore, the combination of SII and other inflammatory indicators, AS-OCT data, and clinical manifestations of patients as evaluation indicators provides a new theoretical basis for the clinical evaluation of pterygium. For patients with high SII value, it can be considered to strengthen the control of immune inflammatory response, which may help to delay the progression of pterygium and indicate the corneal condition after healing.

## Methods

This study was a cross-sectional study. Ninety patients with pterygium in Hebei Eye Hospital from August 2024 to December 2024 were randomly selected and matched with non-pterygium patients. Exclusion criteria included recent (within 1 year) eye surgery, a history of ocular trauma, and ocular inflammatory disease. This study was approved by the Ethical Committee of Hebei Eye Hospital and conformed to the principles of the Declaration of Helsinki. All subjects signed an informed consent form.

**Figure 1:**
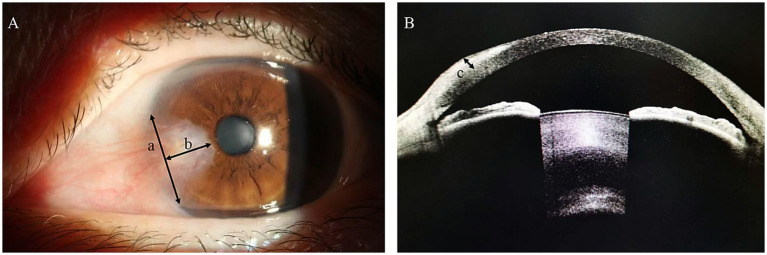
Anterior segment phase and AS-OCT in patients with pterygium.

To assess ocular conditions, all eyes underwent slit-lamp examination, AS-OCT, and anterior segment phase examination, and the Modified Pterygium Classification System was used to score the pterygium patients (4). Diseases (hypertension, diabetes) and signs, complete blood count (neutrophil count, lymphocyte count, monocyte count, platelet count, red blood cell distribution width), and SII, NLR, PLR, and MLR values were evaluated as parameters in this study (15). Measurements were performed using ImageJ software. The lateral distance of the pterygium intrudes on the limbus is defined as a; the vertical distance from the tip of the pterygium to the limbus is defined as b; the thickness of the pterygium on the cornea is defined as c; the area formula is a*b/2; the volume formula is 1/3(c*a*b/2) (Figure 1).

### Statistical analysis

All statistical analyses were performed using SPSS 26.0. The Kolmogorov–Smirnov test was used to evaluate the normality of continuous variables. Non-normal distribution data were described by median (M) and quartile (P25, P75), and the non-parametric rank sum test was used for comparison between groups. Categorical variables were analyzed with the use of the chi-square test or Fisher’s exact test. Ordinal Logistic regression was used to screen all independent variables, and *p* < 0.05 was set as statistically significant. Associations of continuous independent variables were tested by Spearman’s rank correlation.

## Results

In this study, 90 patients with pterygium and 90 control patients were retrospectively analyzed. The clinical characteristics of pterygium patients were analyzed, including 6 cases (6.67%) in group A, 67 cases (74.44%) in group B, and 17 cases (18.89%) in group C. There were 53 females (58.9%) and 37 males (41.1%). The onset time of each group was 2, 5 and 5 months, respectively. The proportion of patients with binocular disease in each group was 0, 23.33, and 14.44%, respectively, with a significant difference (*p* < 0.001) (Table 1).

**Table 1:** Clinical characteristics of patients with pterygium.

(Variables)	A	B	C	*p* value
No. of patients	6 (6.67)	67 (74.44)	17 (18.89)	
Age	64.5 (54,68.5)	64 (57,70)	65 (59,71.5)	0.668
Sex				
Male	1 (1.11)	27 (30)	9 (10)	0.31
Female	5 (5.56)	40 (44.44)	8 (8.89)	
Onset time	2 (1,8.5)	5 (1,10)	5 (3,12.5)	0.298
Monocular/Binocular				
Monocular	6 (6.67)	46 (51.11)	4 (4.44)	**<0.001**
Binocular	0 (0)	21 (23.33)	13 (14.44)	
Outdoor activity time >3 h	4 (4.44)	61 (67.78)	14 (15.56)	0.10
Smoking	0 (0)	13 (14.44)	2 (2.22)	0.61
Drinking alcohol	1 (1.11)	16 (17.78)	2 (2.22)	0.67
Diabetes	0 (0)	3 (3.33)	1 (1.11)	1
Hypertension	1 (1.11)	24 (26.67)	5 (5.56)	0.74

Important findings (*p* < 0.05) are shown in bold. The value of continuous variable is the median (P25, P75), and the dummy variable is the number of cases and proportion (%).

The median values of neutrophil count, monocyte count, SII, NLR, and MLR in pterygium patients (3.73, 0.33, 466.09, 1.92, 0.19) were significantly higher than those in the control group (3.11, 0.30, 402.33, 1.71, 0.17). The differences among the three groups were statistically significant (*p* = 0.005; 0.036; 0.021; 0.029; 0.014). This indicates that compound inflammatory markers were high in blood cells of pterygium patients compared with controls (Table 2).

**Table 2:** Blood cell analysis associated with pterygium development and progression.

Variables	Normal group	Pterygium group	*p* value
Age	59.50 (50.75, 71)	65.00 (57.75, 70.25)	0.072
Neutrophils	3.11 (2.43, 3.92)	3.73 (2.91,4.34)	**0.005**
Lymphocytes	1.88 (1.48, 2.27)	1.78 (1.38, 2.22)	0.377
Platelet count	226.50 (188.50, 259.00)	229.50 (196.50, 29)	0.478
Monocytes	0.30 (0.25, 0.37)	0.33 (0.28, 0.40)	**0.036**
Red blood cell distribution	12.60 (12.20, 13.00)	12.50 (11.90, 12.90)	0.062
SII	402.33 (277.42, 537.83)	466.09 (318.55, 622.83)	**0.021**
NLR	1.71 (1.23, 2.39)	1.92 (1.58, 2.56)	**0.029**
PLR	117.29 (96.39, 150.68)	130.62 (103.11, 160.96)	0.215
MLR	0.17 (0.13, 0.21)	0.19 (0.15, 0.23)	**0.014**

Important findings (*p* < 0.05) are shown in bold. The value of continuous variable is the median (P25, P75), and the dummy variable is the number of cases and proportion (%).

According to the clinical classification of pterygium (the Modified Pterygium Classification System) (4), the patients were divided into three groups: group A (*n* = 6), group B (*n* = 67), and group C (*n* = 17), and the morphological characteristics were further compared. With the increase of corneal grade, the degree of corneal pterygium invasion, the number of blood vessels, and the degree of bulging were increased (*p* < 0.001; *p* < 0.001; *p* = 0.004). The vertical distance of pterygium from the limbus in group A, B and C gradually increased, the horizontal length increased, the thickness of pterygium gradually increased, the area of pterygium increased, the volume of pterygium increased and the weight of pterygium in group B and C became heavier, with statistically significant differences (*p* < 0.001; 0.006; <0.001; <0.001; *p* < 0.001; *p* < 0.001). <0.001; The results showed that there was no significant difference between the two groups (*p* > 0.05) (Table 3).

**Table 3:** Analysis of signs in patients with pterygium.

(Variables)	A6	B67	C17	*p* value
Involvement of cornea				**<0.001**
Level 1	3 (3.33)	0 (0)	0 (0)	
Level 2	3 (3.33)	12 (13.33)	0 (0)	
Level 3	0 (0)	52 (57.78)	5 (5.56)	
Level 4	0 (0)	3 (3.33)	12 (13.33)	
Number of blood vessels				**<0.001**
Level 1	0 (0)	1 (1.11)	0 (0)	
Level 2	6(6.67)	15 (16.67)	1 (1.11)	
Level 3	0 (0)	50 (55.56)	12 (13.33)	
Level 4	0 (0)	1 (1.11)	4 (4.44)	
Uplift				**0.004**
Level 1	3 (3.33)	12 (13.33)	0 (0)	
Level 2	3 (3.33)	48 (53.33)	10 (11.11)	
Level 3	0 (0)	7 (7.78)	6(6.67)	
Level 4	0 (0)	0 (0)	1 (1.11)	
Vertical distance from the limbus	1.32 (1.02, 1.99)	2.69 (1.76, 3.66)	4.00 (3.00, 4.91)	**<0.001**
Transverse plane length	4.56 (3.39, 5.21)	5.15 (4.33, 6.32)	6.34 (5.11, 7.48)	**0.006**
Anterior segment OCT Pterygium thickness	0.31 (0.26, 0.4)	0.44 (0.34, 0.52)	0.52 (0.47, 0.71)	**<0.001**
Area (mm^2^)	3.18 (2.34, 4.16)	7.39 (4.74, 10.72)	11.37 (7.91, 17.80)	**<0.001**
Volume (mm^3^)	0.37 (0.21, 0.51)	1.04 (0.53, 1.42)	1.84 (1.35, 3.73)	**<0.001**
Weight of pterygium (g)	–	0.0035 (0.0025, 0.0045)	0.0073 (0.0041, 0.0092)	**0.024**

Important findings (*p* < 0.05) are shown in bold. The value of continuous variable is the median (P25, P75), and the dummy variable is the number of cases and proportion (%).

The pterygium score was positively correlated with SII, vertical distance from limbus, transverse length, pterygium thickness, pterygium area, pterygium volume, NLR and PLR (R = 0.283, 0.541, 0.492, 0.406, 0.568, 0.663, 0.242, 0.235, respectively). All were statistically significant (*p* < 0.01; <0.01; <0.01; <0.01; <0.01; <0.01; <0.05; <0.05), and the pterygium volume was more strongly correlated with pterygium score than pterygium area (Table 4).

**Table 4:** Spearman correlation study of SII, NLR, PLR, MLR, age, and signs.

Variables	Score	Age	Onset time	SII	NLR	PLR	MLR	Red blood cell distribution	Vertical distance	Transverse plane length	Depth of infiltration	Area	Volume
Score	1												
Age	0.013	1											
Onset time	0.122	−0.135	1										
SII	0.283**	−0.018	0.03	1									
NLR	0.242*	0.077	−0.036	0.803**	1								
PLR	0.235*	−0.171	0.141	0.712**	0.573**	1							
MLR	0.182	0.037	−0.091	0.512**	0.739**	0.524**	1						
Red blood cell distribution	−0.016	−0.104	−0.139	0.055	0	0.082	0.128	1					
Vertical distance	0.541**	0.127	0.026	0.146	0.112	0.148	0.176	−0.072	1				
Transverse plane length	0.492**	0.123	0.047	0.066	0.073	0.074	0.074	0.066	0.683**	1			
Thickness	0.406**	−0.024	−0.04	0.022	0.09	0.082	0.004	0.083	−0.077	0.117	1		
Area	0.568**	0.183	0.102	0.127	0.104	0.161	0.142	−0.046	0.934**	0.850**	0.04	1	
Volume	0.663**	0.133	0.109	0.112	0.116	0.161	0.104	−0.035	0.741**	0.784**	0.424**	0.867**	1

*p < 0.05; **p < 0.01; * * *p < 0.001; Correlation coefficients greater than 0.6 indicate covariance and are shown in bold.

Ordinal logistic analysis was performed on the SII, NLR, PLR, and MLR of patients with pterygium, considering factors such as gender, age, onset time, binocular onset, outdoor time, and pterygium area. In the model constructed with SII as the main component, the OR value of SII was 1.00 (95% CI: 1.00, 1.01) (*p* = 0.008), indicating that for each unit increase in SII, the possibility of pterygium progression would increase by 1.00 times. The OR value of bilateral pterygium was 9.92 (95% CI: 2.03, 48.59) (*p* = 0.005), indicating that the possibility of pterygium progression in bilateral patients was 9.92 times higher than that in unilateral patients. The OR value of pterygium area was 1.29 (95% CI: 1.12, 1.48) (*p* < 0.001), indicating that for every unit increase in pterygium area, the likelihood of pterygium progression would increase by 1.29 times. In the model constructed with NLR as the main component, the OR value of NLR was 2.37 (95% CI: 1.17, 4.79) (*p* = 0.016), indicating that for every unit increase in NLR, the possibility of pterygium progression would increase by 2.37 times. This suggests that SII, bilateral onset, pterygium area, and NLR all significantly increase the risk of pterygium progression. The SII, NLR, PLR, MLR, and the AIC values of the probability model constructed by principal components were 98.97, 100.50, 104.02, and 104.95, respectively, indicating that the SII model was the best one for evaluating the occurrence and development of pterygium (Table 5).

**Table 5:** Ordered logistic of potential biomarkers, pterygium area and progression of pterygium occurrence.

Predictors	OR (95% CI)	*p* value	Predictors	OR (95% CI)	*p* value	Predictors	OR (95% CI)	*p* value	Predictors	OR (95% CI)	*p* value
SII	1.00 (1.00 ~ 1.01)	**0.008**	PLR	1.01 (1.00 ~ 1.03)	0.085	NLR	2.37 (1.17 ~ 4.79)	**0.016**	MLR	180.14 (0.20 ~ 165136.52)	0.136
Gender	1.44 (0.42 ~ 4.97)	0.566	Gender	1.90 (0.57 ~ 6.34)	0.294	Gender	1.46 (0.43 ~ 4.98)	0.545	Gender	1.85 (0.56 ~ 6.11)	0.314
Age	1.01 (0.94 ~ 1.08)	0.829	Age	1.02 (0.95 ~ 1.09)	0.672	Age	1.00 (0.93 ~ 1.07)	0.992	Age	1.00 (0.93 ~ 1.07)	0.966
Onset time	1.01 (0.96 ~ 1.07)	0.655	Onset time	1.00 (0.95 ~ 1.05)	0.912	Onset time	1.02 (0.97 ~ 1.07)	0.553	Onset time	1.01 (0.96 ~ 1.06)	0.652
Monocular/binocular	9.92 (2.03 ~ 48.59)	**0.005**	Monocular/binocular	10.94 (2.42 ~ 49.57)	**0.002**	Monocular/binocular	14.18 (2.82 ~ 71.25)	**0.001**	Monocular/binocular	13.26 (2.89 ~ 60.96)	**0.001**
Outdoor activity time >3 h	2.84 (0.53 ~ 15.20)	0.224	Outdoor activity time >3 h	1.89 (0.39 ~ 9.07)	0.426	Outdoor activity time >3 h	1.91 (0.40 ~ 9.20)	0.421	Outdoor activity time >3 h	1.82 (0.37 ~ 8.92)	0.458
Area	1.29 (1.12 ~ 1.48)	**0**	Area	1.25 (1.09 ~ 1.43)	**0.001**	Area	1.28 (1.12 ~ 1.46)	**0**	Area	1.26 (1.11 ~ 1.44)	**0.001**

SII = platelet * (neutrophils/lymphocytes), PLR = blood/lymphocytes, NLR = medium/lymphocytes, MLR = single/lymphocytes. Data with *p*<0.05 are shown in bold. * represents multiplication.

Ordinal logistic analysis was performed on the SII, NLR, PLR, and MLR of patients with pterygium, considering factors such as gender, age, onset time, binocular onset, outdoor time, and pterygium volume. In the model constructed with SII as the main component, the OR value of SII was 1.01 (95% CI: 1.00, 1.01) (*p* = 0.008), indicating that for each unit increase in SII, the possibility of pterygium progression would increase by 1.01 times. The OR value of bilateral pterygium was 7.20 (95% CI: 1.21, 43.05) (*p* = 0.03), indicating that the possibility of pterygium progression in bilateral patients was 7.20 times higher than that in unilateral patients. The OR value of pterygium volume was 9.29 (95% CI: 2.90, 29.76) (*p* < 0.001), indicating that for every unit increase in pterygium volume, the likelihood of pterygium progression would increase by 9.29 times. In the model constructed with NLR as the main component, NLR had an OR value of 2.29 (95% CI: 1.04, 5.06) (*p* = 0.04), indicating that for every unit increase in NLR, the possibility of pterygium progression increased by 2.29 times. This suggests that SII, bilateral onset, pterygium volume, and NLR all significantly increase the risk of pterygium progression. The SII, NLR, PLR, MLR, and AIC values of the probability model constructed by principal components were 85.52, 89.45, 91.88, and 92.95, respectively, indicating that the SII model was the best one for evaluating the occurrence and development of pterygium (Table 6).

**Table 6:** Ordered logistic of potential biomarkers, pterygium volume, and progression of pterygium occurrence.

Predictors	OR (95% CI)	*P* Value	Predictors	OR (95% CI)	*p* value	Predictors	OR (95% CI)	*p* value	Predictors	OR (95% CI)	*p* value
SII	1.01 (1.00–1.01)	**0.008**	PLR	1.01 (1.00–1.03)	0.136	NLR	2.29 (1.04–5.06)	**0.04**	MLR	112.00 (0.03–399152.24)	0.258
Gender	1.43 (0.35–5.78)	0.616	Gender	2.04 (0.56–7.49)	0.281	Gender	1.64 (0.43–6.30)	0.471	Gender	2.04 (0.56–7.44)	0.283
Age	1.01 (0.94–1.09)	0.753	Age	1.02 (0.94–1.10)	0.674	Age	1.01 (0.94–1.08)	0.866	Age	1.00 (0.93–1.08)	0.921
Onset time	1.01 (0.95–1.07)	0.846	Onset time	1.00 (0.95–1.06)	1	Onset time	1.01 (0.96–1.07)	0.73	Onset time	1.01 (0.96–1.06)	0.739
Monocular/binocular	7.20 (1.21–43.05)	**0.03**	Monocular/binocular	10.63 (1.93–58.69)	**0.007**	Monocular/binocular	12.01 (2.07–69.78)	**0.006**	Monocular/binocular	12.84 (2.33–70.89)	**0.003**
Outdoor activity time >3 h	2.03 (0.33–12.52)	0.444	Outdoor activity time >3 h	1.21 (0.22–6.66)	0.823	Outdoor activity time >3 h	1.24 (0.23–6.81)	0.805	Outdoor activity time >3 h	1.16 (0.21–6.52)	0.866
Volume	9.29 (2.90–29.76)	**0**	Volume	7.06 (2.43–20.49)	**0**	Volume	7.55 (2.64–21.61)	**0**	Volume	7.12 (2.54–19.97)	**0**

SII = platelet * (neutrophils/lymphocytes), PLR = blood/lymphocytes, NLR = medium/lymphocytes, MLR = single/lymphocytes. Data with *p*<0.05 are shown in bold. *represents multiplication.

## Discussion

Pterygium, a fibrovascular tissue that grows from the bulbar conjunctiva to the cornea, is a common ocular surface lesion (16). The harm of pterygium is mainly reflected in the concealment of its course and the uncertainty of its progress (17). It is often difficult to detect in the early stages of the disease. When the symptoms are obvious, pterygium has involved a large area of the cornea, affecting the visual acuity after recovery. In addition, the progression rate of pterygium is variable, and it is difficult for patients to judge by themselves, which often leads to delayed treatment. Traditionally, the severity assessment of pterygium mainly relies on the clinical classification under the slit lamp. This classification is simple but subjective, and it is difficult to accurately quantify the three-dimensional morphology and biological activity of the lesion (18). The combination of systemic inflammatory markers and pterygium morphological parameters obtained by AS-OCT is of great significance for understanding the occurrence and development of pterygium and for early intervention and treatment of patients.

SII is a good biomarker of local immune response and systemic inflammatory response, which can be used to predict prognosis and has been confirmed to be a good biomarker for a variety of tumors (19). One of the pathological features of pterygium is the infiltration of inflammatory cells (17). Previous studies have shown that SII expression is increased in patients with pseudoexfoliation syndrome, pseudoexfoliation glaucoma, dry eye, and endophthalmitis, and can predict the progression of diabetic retinopathy (20–23). In eyelid tumors, high levels of NLR and SII can be used as auxiliary indicators to judge benign or malignant tumors (24). The present study found that patients with pterygium exhibited significantly higher neutrophil count, monocyte count, and SII, NLR, and MLR values compared to controls, indicating that inflammatory markers accurately described the inflammatory response in blood cells in the pterygium patient group. This finding is consistent with existing clinical studies on systemic inflammatory markers and pterygium (25). SII and other composite indicators comprehensively reflect the balance between neutrophil pro-inflammation and lymphocyte immune regulation. The increase of SII suggests that there is a persistent and systemic pro-inflammatory environment in the patient’s body, which may be a key factor driving abnormal hyperplasia, angiogenesis and fibrosis of local ocular surface tissues.

On the basis of revealing the relationship between systemic inflammation and pterygium, 90 patients with pterygium were divided into three groups (A, B, and C) according to the clinical classification (the Modified Pterygium Classification System) (4). The relationship between the level of systemic inflammation and the morphological parameters of pterygium, such as corneal invasion distance, thickness, area, volume, and actual weight, was systematically explored. Pterygium volume was counted for the first time in a cross-sectional study by AS-OCT. The correlation analysis showed that the pterygium score was positively correlated with SII and multiple pterygium morphological indicators. Among them, the correlation between pterygium volume and clinical scores was stronger than that of pterygium area, indicating that the pterygium volume, as a three-dimensional index, may be better than the traditional two-dimensional parameters in evaluating the severity of pterygium.

The increase of vertical distance, transverse length, depth of pterygium, and volume of pterygium are the direct manifestations of pathological processes such as cell proliferation, extracellular matrix deposition, and vascular invasion in the tissue (18). It has been proven that chronic inflammation can promote the imbalance of the microenvironment of limbal stem cells, and induce the activation of fibroblasts, neovascularization, and collagen deposition, thereby accelerating the invasion of pterygium into the central cornea, leading to local uplift and irregular curvature (16, 26–28). The findings suggest that this process is synchronized with an increase in systemic inflammation levels.

In order to clarify the independent role of related factors in disease progression, an ordinal Logistic regression model was constructed. We found that SII and binocular onset were independent risk factors for pterygium progression in both models of pterygium area and pterygium volume, suggesting a significant association of SII with disease progression. In addition, among the four inflammatory indicators of SII, NLR, PLR, and MLR, the model based on SII had the best fit, indicating that SII is a more stable and efficient predictor than NLR and other traditional inflammatory indicators in assessing the risk of pterygium. This may be because SII covers the three pathways of neutrophils, lymphocytes, and platelets at the same time, which can more comprehensively capture the inflammation-immune-thrombosis network state of the body (20). The OR value of the pterygium volume model was higher than that of the pterygium area, indicating that the risk of disease progression per unit volume increase is much higher than that per unit area. Therefore, the pterygium volume measured by AS-OCT can be included in the clinical evaluation system, which can identify the individuals who are susceptible to progression more sensitively and provide a more accurate quantitative basis for clinicians to determine when to intervene.

However, our study has some limitations. Firstly, this is a single-center cross-sectional study, and the causal relationship between SII and pterygium progression cannot be established, which needs to be verified by prospective cohort studies in the future. Second, the sample size was relatively limited, especially since the small number of samples in group A may have introduced bias. Finally, the lack of detection of local inflammatory factors in the tear or pterygium tissue could not directly confirm the causal relationship between peripheral blood inflammatory indicators and local inflammatory responses. In the future, large sample and prospective cohort studies are needed to further verify the predictive value of SII and explore its potential mechanism combined with molecular biology methods.

In summary, this study proposes a new paradigm for the assessment of pterygium: combining the systemic inflammatory marker SII with AS-OCT morphometry. SII is a powerful predictor of the occurrence and progression of pterygium. Compared with the pterygium area, pterygium volume can more accurately evaluate the severity and progression of the disease. This assessment strategy helps clinicians to identify patients with rapidly progressing pterygium earlier and more accurately, leading to more individualized treatment.

## Data Availability

The original contributions presented in the study are included in the article/supplementary material, further inquiries can be directed to the corresponding authors.
